# Armut, Hunger, Hilfe: Ein Blick auf Fragen von Armut und Ernährung im Kontext der Sozialen Arbeit

**DOI:** 10.1007/s12592-022-00441-5

**Published:** 2022-12-20

**Authors:** Holger Schoneville

**Affiliations:** Fakultät für Erziehungswissenschaft, Universität Hamburg, Von-Melle-Park 8, 20146 Hamburg, Deutschland

**Keywords:** Armut, Hunger, Armutshilfe, Suppenküchen, Tafeln, Poverty, Hunger, Assistance, Soupkitchen, Food banks

## Abstract

Vor dem Hintergrund der aktuellen Steigerungen der Kosten des alltäglichen Lebens, insbesondere auch im Bereich der Ernährung, beschäftigt sich der Beitrag mit Fragen von Armut und Essen im Kontext der Sozialen Arbeit. Dabei wird der Versuch einer Einordnung und Diskussion von Hilfsangeboten im Feld der Sozialen Arbeit vorgenommen, die als Ernährungshilfen verstanden werden können. Der Beitrag zielt auf eine Einordnung des Feldes sowie kritische Diskussion der Armutshilfen und ringt dabei um eine professionspolitische Positionierung.

## Abstract (en)

In light of the current cost of living crisis, this article focuses on issues of poverty and food in the context of social work. It will systematise and discuss different forms of assistance in the field of social work which can be understood as food assistance. The contribution aims at a systematisation of the field as well as a critical discussion of poverty assistance.

„Heizung abstellen oder weniger essen? Die Ärmsten sollten sich nicht entscheiden müssen“ (Tagesspiegel, 16.07.2022)^1^

„Arm durch Inflation: Wie Preissteigerungen zum Risiko werden“ (NDR, 06.09.2022)

„Inflation lässt Münchner Rentner verzweifeln: ‚Dann muss ich hungern“ (Münchener Merkur, 04.10.2022)

Die aktuellen Schlagzeilen in den Medien weisen auf die drastischen Folgen der steigenden Kosten für den allgemeinen Lebensunterhalt hin. Die Preissteigerungen zeigen sich im konkreten Lebensalltag und treffen jene Menschen besonders hart, die schon zuvor von Armut oder Prekarität betroffen waren. Auch medial werden soziale Existenzängste und Sorgen um die grundlegende Versorgung thematisiert. Damit rücken nicht nur Fragen der ungleichen Verteilung auf die Tagesordnung, sondern in auch die Lebensrealität von Menschen, deren ökonomische Ressourcen so limitiert sind, dass sie erheblich in ihrem Alltag eingeschränkt sind. Deutlicher formuliert: Wir sind auf neue Weise mit Armutsfragen konfrontiert.

Innerhalb des Beitrags wird die aktuelle Situation zum Anlass genommen, Fragen von Essen und Armut im Kontext der Sozialen Arbeit zu betrachten. In einem ersten Schritt wird dazu ein Blick auf die aktuelle Krise der Lebensunterhaltskosten geworfen und deren Bedeutung für Armut und Ernährung betrachtet. Im Zentrum des Beitrags stehen dann jedoch Angebote der Sozialen Arbeit, die auf unterschiedliche Weise auf Fragen von Ernährung im Kontext von Armut reagieren. Dazu werden unterschiedliche Ernährungs- und Versorgungsangebote der Sozialen Arbeit im Kontext von Armutslinderung und Armutsbekämpfung diskutiert. Der Beitrag zielt auf eine Einordnung dieser Angebotsformen im Kontext einer kritischen Diskussion um Armutshilfen und ringt dabei um eine professionspolitische Positionierung für die Soziale Arbeit.

## Armut, Inflation, Ernährung

Dass Menschen in Deutschland von Armut betroffen sind, obwohl die deutsche Gesellschaft eine der reichsten Gesellschaften der Welt ist, ist nicht neu. Jedoch ist die derzeitige Situation sowohl hinsichtlich der Qualität als auch der Quantität neuartig. Die Lebensrealitäten verschärfen sich drastisch, dies betrifft vor allem Menschen, die unter den Bedingungen von Armut ihr Leben realisieren müssen. Aber auch das quantitative Ausmaß der von Armut direkt betroffenen oder bedrohten Personengruppen erhöht sich. Aktuelle Berechnungen zeigen auf, dass die Belastungen durch die Inflation für Gruppen mit geringem Einkommen besonders hoch sind (Deutsches Institut für Wirtschaftsforschung 2022).

**Figure 1:**
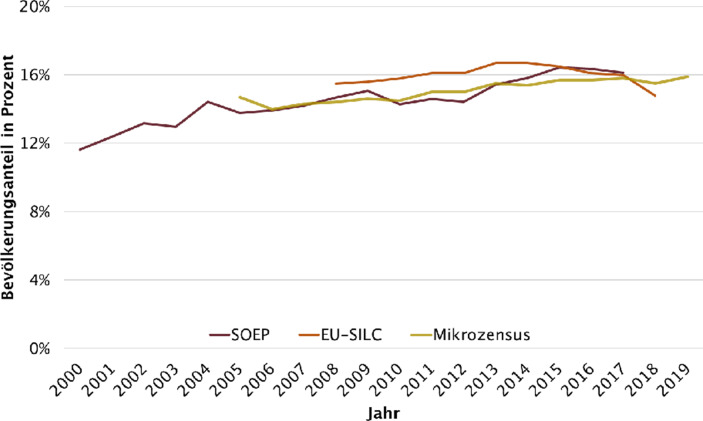

Diese Entwicklung trifft auf eine Grundsituation, in der schon bislang eine große Gruppe von Armut betroffen waren: Der letzte Armuts- und Reichtumsbericht der Bundesregierung zeigte nochmals, dass die Armutsrisikoquote seit Anfang des Jahrtausendwechsels von 11,7 % (im Jahr 2000) auf 15,9 (im Jahr 2018) anstieg^2^ (Bundesministerium für Arbeit und Soziales 2021; Abb. 1). Eine zentrale Botschaft des Berichts lag in dem empirischen Nachweis, dass sich die Armutslagen verfestigt haben. Im Kern bedeutet dies also, dass sich von Armut betroffene Menschen immer seltener aus diesen Lagen befreien können.

Zu beachten ist dabei, dass sich die empirischen Befunde auf die vergangenen Jahre beziehen und somit die aktuellen gesellschaftlichen Veränderungen noch nicht abbilden. Die gegenwärtige Armutsbetroffenheit wird sich durch die Inflation somit deutlich verschärfen. Dies gilt erstens für Menschen, die schon vorher von Armut betroffen waren, darüber hinaus jedoch auch für Menschen, die bislang unter Bedingungen gelebt haben, die als „prekärer Wohlstand“ (Hübinger 1996) verstanden werden können. Drittens sind darüber hinaus gesellschaftliche Gruppen bis zur unteren Mittelschicht insofern betroffen, als sie Erfahrungen der Prekarisierung machen werden und sich Zukunftsängste dieser Gruppen verstärken (Nachtwey 2016).

**Figure 2:**
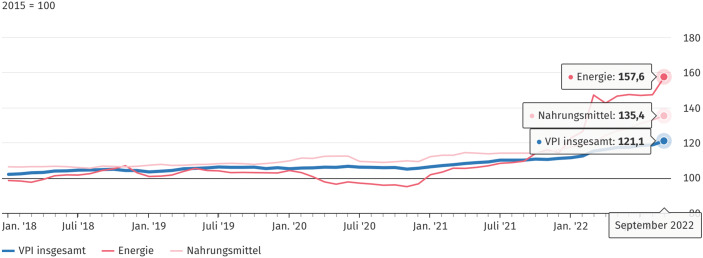

Zu den Bereichen, in den denen die Inflation am stärksten auf die Lebensrealität wirkt, gehören die Versorgung der Haushalte mit Strom und Gas, die gestiegenen Preise für Diesel und Benzin sowie die Kosten im Bereich der Ernährung (Abb. 2).

Wohlhabendere Gruppen haben die Möglichkeit, auf diese Teuerungen durch die Umstellung ihrer Lebens- und Konsumgewohnheiten zu reagieren: Die Heizung wird später angeschaltet, über die Installation von Solarenergie auf dem Dach nachgedacht, Restaurantbesuche reduziert, Backwaren werden nicht mehr in der Bäckerei, sondern beim Discounter gekauft. Von Armut betroffene Gruppen haben diese Einsparmöglichkeiten nicht, da sie schon vorher auf diese Dinge verzichten mussten. Sie trifft die Preissteigerung in ihrer grundlegenden Versorgung.

Anschaulich wird dies vor allem bei Empfänger*innen von Arbeitslosengeld-II-Leistungen: Das ALG-II ist so konzipiert, dass es den Lebensunterhalt sichern soll und geht dabei von Bedarfen im Bereich der Ernährung, Kleidung, Körperpflege, Hausrat, Haushaltsenergie und persönlichen Bedürfnisse aus^3^. Für diese Bedarfe wird von einem Regelbedarf ausgegangen und dieser wiederum als monatliche Geldleistung berechnet: Für eine alleinstehende Person wurde so ein Regelbedarf von insgesamt 449 € im Monat festgesetzt. Hinzu kommt die Übernahme von angemessenen Kosten für Unterkunft und Heizung. Von den 449 € entfallen auf den Bereich der Ernährung derzeit 155,82 €/Monat, für Wohnen, Energie, Wohninstandhaltung wird von 38,07 €/Monat ausgegangen und der Bereich Verkehr mit 40,27 €/Monat kalkuliert. Schon bislang wurde kritisiert, dass diese Regelsätze, vor allem auch im Bereich der Ernährung mit umgerechnet circa 5,20 € pro Tag, zu knapp berechnet seien und nicht dem Bedarf genügen (für den Paritätischen Wohlfahrtsverband so z. B. Aust 2021). Aber auch unabhängig davon wird deutlich, dass die erheblichen Preissteigerungen für Lebensmittel davon nicht zu decken sind.^4^ Die Gefährdung des existenziellen Minimums zeigt sich bei Empfänger*innen von ALG-II-Leistungen besonders eindrücklich, geht aber über diese hinaus und betrifft auch Gruppen mit Erwerbseinkommen, die knapp oberhalb der Grenzen für Unterstützungsleistungen liegen.

Dass die grundlegende Ernährung für einige von Armut betroffenen Personen nicht sichergestellt ist, spielte bislang zumindest innerhalb der offiziellen Berichterstattung zum Thema Armut keine zentrale Rolle. Innerhalb des Armuts- und Reichtumsberichts der Bundesregierung finden sich zwar Hinweise zur Ernährung, diese beziehen sich jedoch vor allem auf Ernährungsweisen, welche innerhalb des Berichts als problematisch markiert und speziell gesellschaftlichen Gruppen zugeordnet werden, die unter den Bedingungen von Armut leben. Erst kürzlich kritisierte Martin Rücker (2022) die im Armuts- und Reichtumsbericht vorgenommene Analyse, indem er aufzeigte, dass die strukturelle Bedeutung von Armut in Bezug auf Ernährung verkürzt dargestellt wird.

Vor diesem Hintergrund verwundert es nicht, dass Sabine Pfeiffer den Hunger ganz allgemein als „ein unerwartetes Thema in der Überflussgesellschaft“ (Pfeiffer 2014, S. 5) darstellt. Sie zeigt dabei, dass Ernährungsarmut und Hunger in Deutschland häufiger vorkommen und verweist dabei u. a. auf eine Studie von Christine Kaiser (2001), die schon zu Zeiten der Sozialhilfe zeigte, dass bis zu 70 % der Sozialhilfeempfänger*innen beim Essen sparen und bei zwei Dritteln das Budget nicht für eine bedarfsgerechte Ernährung ausreicht (Kaiser 2001, S. 48 ff.). Insgesamt beschreibt Pfeiffer (2014) Ernährungsarmut damit als eine verdrängte Realität in der deutschen Gegenwartsgesellschaft. Diese verdrängte Realität ist auch – und vielleicht sogar besonders – für die Soziale Arbeit von zentraler Bedeutung.

## Armut und Essen im Kontext der Sozialen Arbeit

Soziale Arbeit als moderne personenbezogene Dienstleistungsarbeit wird, auch wenn sie als Profession und Disziplin immer wieder mit Fragen von Armut konfrontiert ist, eher selten mit Hunger und Ernährungsarmut in Verbindung gebracht. Dabei liegt die Verbindung zur Sozialen Arbeit näher, als dies zunächst erscheinen mag. Dies liegt nicht nur daran, dass Fragen von Essen und Ernährung im pädagogischen Alltag eine zentrale Rolle spielen. Vielmehr war für die Vorläufer der heutigen modernen Sozialen Arbeit armutsbedingter Mangel im Bereich der Ernährung und Versorgung ein zentrales Problem, auf welches mit entsprechenden Hilfsangeboten reagiert wurde. In diesem Sinne beschreiben Lotte Rose und Benedikt Sturzenhecker „Ernährungsnotlagen als Wurzel der Profession“ (2009, S. 10). Sie weisen damit auf die historische Bedeutung der Versorgung mit Nahrung im Kontext der Armenfürsorge hin und formulieren dabei: Während im 19. Jahrhundert grundlegende Ernährungsnotlagen bestanden, die zu Hungersnöten und -aufständen führten, sei die Armenfürsorge „immer auch […] mit der Aufgabe verbunden [gewesen, H.S.], bedürftige Menschen mit der notwendigen Nahrung zu versorgen“ (Rose und Sturzenhecker 2009, S. 10). Auch Christine Meyer (2021) weist darauf hin, dass die Soziale Arbeit konstitutiv mit Essen verbunden sei: zum einen im Allgemeinen, weil Essen in den meisten pädagogischen Settings eingewoben ist (Meyer 2018); zum anderen jedoch auch historisch über die Verbindung zur Armut und Armutspolitiken, als Phänomene von Hunger (Meyer 2021). Über die historische Dimension hinaus geht Meyer dabei davon aus, dass es „auch in nahrungssicheren Gesellschaften, vielleicht sogar eher ‚Überflussgesellschaften‘ wiederkehrend einen Anteil an Menschen [gibt, H.S.], der Hungererfahrungen gemacht hat und auf die Soziale Arbeit in ihrer täglichen Arbeit trifft“ (Meyer 2021, S. 36). Sie verbindet dies mit einem Plädoyer an die Professionalität in den Handlungsfeldern der Sozialen Arbeit und formuliert, dass es zu den täglichen Aufgaben in der Sozialen Arbeit gehöre, dass Nahrungsmangel richtig erkannt wird und angemessene Reaktionen gefunden werden.

Aufbauend auf dieser grundsätzlichen Verortung von Ernährung und Hunger im Kontext der Sozialen Arbeit, sollen im Folgenden einige Essens- und Ernährungsangebote als Armutshilfen im Kontext der Sozialen Arbeit diskutiert werden.

### Essensangebote in pädagogischen Einrichtungen – unterschätzte Armutshilfe?

Die Mittagsangebote in Schulen, die Verpflegung in Heimen, das Essen in Kitas, die Versorgung im Bereich der Altenhilfe, der Snack in der Jugendgruppe, das gemeinsames Kochen im Jugendzentrum und viele andere mehr sind integrale Bestandteile des Alltags in den Einrichtungen der Sozialen Arbeit (vgl. die Beiträge in Täubig 2016; Rose und Sturzenhecker 2009; Schulz et al. 2020). Essensangebote stehen in der Regel allen Adressat*innen der jeweiligen Einrichtungen offen und fokussieren somit nicht armutsbetroffene Menschen. In ihrer Bedeutung für Menschen in Armut werden die regulären Essensangebote in Sozial- und Bildungseinrichtungen vermutlich deutlich unterschätzt.

Anekdotische Berichte aus Schulen, Jugendzentren und anderen Einrichtungen, in denen dargestellt wird, dass ein Teil der Kinder und Jugendlichen hungrig in die Einrichtungen kämen und die jeweiligen Essensangebote von zentraler Bedeutung seien (zum Beispiel bei Rost 2010), verweisen jedoch bereits darauf, dass diese Angebote eine wichtige Rolle spielen. Darüber hinaus werden im Kontext der Debatte um Ganztagsbetreuung in der Kooperation zwischen Schule und Jugendarbeit (kostenlose) „Ernährungsangebote als Reaktion auf die Armut von Kindern und Jugendlichen“ (Deinet 2009, S. 127) diskutiert. Besonders deutlich wurde dies kürzlich in Großbritannien, wo in der ersten Hälfte des Jahres 2020 im Zuge der „Lockdown“-Maßnahmen zur Bekämpfung der COVID-19-Pandemie Schulen geschlossen wurden. In Großbritannien sind jedoch 1,3 Mio. Kinder und Jugendliche für das kostenlose Schulessen registriert, und für circa ein Drittel dieser Kinder und Jugendlichen sollte das Essen ersatzlos entfallen. Gegen Widerstand der konservativen Regierung unter der Führung von Boris Johnson bildete sich Protest von unterschiedlichen Initiativen. Besonders hervorgetreten ist dabei der britische Profifußballer Marcus Rashford, der sich unter anderem mit einem öffentlichen Brief an das Parlament und die Regierung wendete (Rashford 2020).

Auch wenn sich die Relevanz dieser regulären, integrierten Essensangebote in Bezug auf ihre konkreten Effekte im Kontext von Armutsbekämpfung nur schwer bestimmen lässt, ist anzunehmen, dass gerade die Integration von kostenlosen oder günstigen Mahlzeiten in den Alltag der Einrichtungen eine Großzahl von armutsbetroffenen Menschen erreichen kann. Einen Beitrag zur Klärung dieser Frage könnten empirische Forschungsprojekte leisten, die danach fragen, welche Bedeutung diese allgemeinen Essensangebote für die Alltagsbewältigung von armutsbetroffenen Menschen (insbesondere Familien) haben.

### Suppenküchen und Co. – Essensausgaben als Nothilfen

Innerhalb der Sozialen Arbeit gibt es eine lange Tradition von unterschiedlichen Formen der Essensausgaben als Nothilfen. Sie gehören zur Geschichte der Armenfürsorge (Sachße und Tennstedt 1980). Im Zuge der Entstehung wohlfahrtsstaatlicher Sicherungssysteme haben diese Essenshilfen an Bedeutung verloren, sind aber nicht vollständig verschwunden. Vielmehr richten sie sich heute mit ihren Angeboten vor allem an Gruppen, die in existenzieller Weise von Armut betroffen sind und oftmals nicht von anderen Hilfeformen erreicht werden können. So sind sie etwa genuiner Bestandteil der modernen Obdach- und Wohnungslosenhilfe.

Stefan Gillich und Rolf Keicher (2016a) schlagen vor, sie als Teil von verschiedenen Formen der Überlebenshilfen und existenziellen Absicherung zu verstehen: „Dazu gehört die Bereitstellung von Übernachtungsmöglichkeiten, die Möglichkeit zu duschen und Wäsche zu waschen oder die Einrichtung einer Postadresse. Ganz handfest sind damit aber auch reale Essensangebote in Tagestreffs oder in anderen niederschwelligen Angebotsformen gemeint, die einen Einstieg in ein planmäßiges Hilfeverfahren erleichtern können oder Menschen vor dem Verhungern oder Erfrieren bewahren“ (Gillich und Keicher 2016a, S. 10). Zugleich sind sie immer auch Orte der Begegnung und Sozialität (Reidegeld und Reubelt 1996, S. 9).

Im vorstehenden Zitat formulieren Gillich und Keicher (2016a) bereits einen Anspruch, den sie mit diesen Formen der Nothilfe verknüpfen: Sie gehen davon aus, dass die Angebote zwar konkrete existenzielle Versorgung leisten, aber auch darüber hinaus zielen müssen. Sie weisen darauf hin, dass die Angebote im Zusammenhang mit planmäßigen Hilfeverfahren zu sehen sind. Dies bedeutet nicht, dass die Essenshilfen für die Nutzer*innen an Bedingungen geknüpft werden. Konzeptionell gehen die Essenshilfen jedoch über die Versorgung hinaus und zielen auf die Ermöglichung von Hilfsangeboten, welche die Lebenssituation der betroffenen Menschen grundlegend verbessern können. In dem von Stefan Gillich und Rolf Keicher (2016b) vorgelegten Herausgeberband wird dies unter den Stichworten „Suppe, Beratung, Politik“ diskutiert. Damit geht die Forderung einher, dass akute Überlebenshilfe, die unter der Metapher „Suppe“ gefasst wird, an Formen der „Beratung“ geknüpft sein müssen, die über die akute Überlebenshilfe hinausgehen. Mit „Politik“ wird darauf hingewiesen, dass die konkreten Problemlagen und Hilfsmöglichkeiten immer auch politischer Natur sind und somit entsprechend reflektiert werden müssen. Soziale Arbeit wird damit insgesamt als politische und parteiliche Sozialarbeit entworfen (Gillich 2016).

### Lebensmittelausgaben der „Tafeln“ – Symbol einer neuen Mitleidsökonomie

Wenige Akteure im Feld der Sozialen Arbeit standen in den vergangenen Jahren so im Licht der Öffentlichkeit, wie dies bei den sogenannten „Tafeln“ der Fall ist. Die Lebensmittelausgaben beschreiben sich selbst als eine direkte Reaktion auf Armutsfragen (vgl. Wunderlich 2013; Ehlert 2013; Bruckdorfer 2013). Sie charakterisieren sich als die „größte sozial-ökonomische Bewegung in Deutschland, die Lebensmittel rettet und an armutsbetroffene Menschen weitergibt“ (Tafel Deutschland 2022a).^5^ Sie sammeln Lebensmittel ein, die im regulären Handel keine Verwendung mehr finden und vernichtet würden und organisieren die Verteilung dieser an von Armut betroffene Personen. Die Hilfeleistungen beruhen somit auf freiwilligen Gaben. Dies trifft sowohl auf die gespendeten Produkte zu als auch auf die dort tätigen Ehrenamtlichen, die ihre Zeit spenden. Nach den Zahlen des Bundesverbands (Tafel Deutschland 2022c, b) nutzen derzeit zwischen 1,65 und 2 Mio.^6^ armutsbetroffene Menschen die Angebote der über 2000 lokalen Ausgabestellen. Markus Grabka und Jürgen Schrupp (2022) kommen in einer kürzlich veröffentlichten Studie des Deutschen Instituts für Wirtschaftsforschung (DIW) zu dem Ergebnis, dass im Jahr 2020 1,1 Mio. Menschen die Lebensmittelausgaben genutzt haben.^7^

Auch wenn zuweilen infrage gestellt wird, ob die Lebensmittelausgaben überhaupt zur modernen, professionalisierten Sozialen Arbeit zu zählen sind, sprechen mindestens drei Gründe dafür, sie als Teil der Sozialen Arbeit zu begreifen: Ein erster Grund liegt darin, dass sie in einer Traditionslinie der Armenfürsorge stehen bzw. frühe Formen dieser Linie aufgreifen. Sie reagieren auf Armutsprobleme, indem sie Hilfsangebote im Bereich der Versorgung mit Lebensmitteln anbieten und ergänzen diese an vielen Stellen durch weitere Angebote. Ein zweiter Grund liegt darin, dass die Nutzer*innen häufig gleichzeitig auch Adressat*innen der Sozialen Arbeit darstellen, nicht selten sind sie sogar in der ein oder anderen Weise Nutzer*innen von konkreten Angeboten professioneller sozialer Dienstleistungen. Schließlich werden, drittens, die Lebensmittelausgaben zum großen Teil von den gleichen Akteur*innen getragen wie die etablierten Angebote der Sozialen Arbeit. So weist der Bundesverband der Tafeln aus, dass die Mehrheit (60 %) der in ihr organisierten Lebensmittelausgaben einem der etablierten Wohlfahrtsverbände angehören (Tafel Deutschland 2022c).

In der Zwischenzeit sind auch Positionen zu vernehmen, die für eine Professionalisierung der Lebensmittelausgaben plädieren und dabei Parallelen zur Geschichte der Sozialen Arbeit insgesamt ziehen (Dietz und Wegener 2021). Sie gehen davon aus, dass die Soziale Arbeit in ihren Anfängen ebenfalls von zivilgesellschaftlichen Initiativen getragen wurde, ehrenamtliches Engagement zentral war und sich erst über die Zeit eine institutionalisierte und professionalisierte Soziale Arbeit herausgebildet habe. Gegen diese Argumentation ist jedoch kritisch einzuwenden, dass die Anfänge der Sozialen Arbeit vor der Institutionalisierung des Wohlfahrtsstaats lagen bzw. sich parallel zu dieser entwickelt haben (Kessl und Schoneville 2021). Die Lebensmittelausgaben und ähnliche Angebote haben sich jedoch in einer gesellschaftlichen Konstellation herausgebildet, in der ein entwickeltes System sozialstaatlicher Sicherung vorherrscht.

Aus einer sozialstaatstheoretischen Perspektive lässt sich beobachten, dass die Lebensmittelausgaben und ähnliche Angebote im Kontext eines umfassenden Transformationsprozesses des wohlfahrtsstaatlichen Arrangements insgesamt zu verstehen sind. Dieser Veränderungsprozess ist durch die Infragestellung und Neugestaltung sozialstaatlicher Sicherungssysteme geprägt und ging im Bereich der Politik zur Armutsbekämpfung mit einem Abbau von Leistungen einher. Innerhalb dieses gesellschaftlichen Kontextes haben sich die Lebensmittelausgaben und ähnliche Angebote für von Armut betroffene Menschen etabliert. Die spendenbasierten Lebensmittelausgaben sind dabei in eine Lücke getreten, welche die staatlichen sozialen Sicherungssysteme hinterlassen haben. Sie können als „Schatten des Sozialstaats“ (Kessl und Schoneville 2021, 2010) verstanden werden und sind damit ein funktionaler Teil des wohlfahrtsstaatlichen Arrangements (Schoneville 2018). Sie stehen damit symptomatisch für den Wandlungsprozess des Sozialstaats insgesamt. Aktuell zeichnet sich eine noch stärkere Verschmelzung der zwischen den Lebensmittelausgaben und dem Staat ab: Dies zeigt sich unter anderem daran, dass im Zuge der COVID-19-Krise der Bundesverband der deutschen „Tafeln“ erstmals öffentlich staatliche Unterstützung eingefordert hat, um die erhöhte Nachfrage nach Unterstützung zu bewältigen (Tafel Deutschland 2020). Mittlerweile gibt es vermehrt solche Formen der staatlichen Unterstützung, insbesondere in Form von Modellprojekten. Dies ist eine entscheidende Veränderung, da in der Bundesrepublik Deutschland die Trennung zwischen den zivilgesellschaftlichen Angeboten der Lebensmittelausgaben und den staatlichen Unterstützungsleistungen bislang besonders betont wurde. Im Rahmen eines internationalen Vergleichs konnte gezeigt werden, dass die direkte Unterstützung in beinahe allen anderen Ländern der Europäischen Union schon länger und deutlich ausgeprägter ist (Greiss et al. 2022), wobei insbesondere Mittel aus dem EU Fund for European Aid to the Most Deprived (FEAD) eine bedeutende Rolle zukommt (Greiss und Schoneville 2022). Insgesamt zeigt sich darin, dass die Lebensmittelhilfen zum integralen Bestandteil des wohlfahrtsstaatlichen Arrangements werden und die Grenzen zwischen staatlichem und zivilgesellschaftlichem Angebot verschwimmen. Dies passiert jedoch, ohne dass dadurch gleichzeitig staatlich garantierte Rechte auf Unterstützung entstehen. Vielmehr werden die (teil)staatlichen Akteure hier zu Quasi-Charity-Organisationen.

Im Kontext der sozialpädagogischen Diskussion betonen vor allem Positionen aus der Adressat*innen- und Nutzer*innenforschung (vgl. u. a. Bitzan und Bolay 2013; Oelerich und Schaarschuch 2013) die Bedeutung der Perspektive der Adressat*innen und Nutzer*innen selbst – insbesondere auch hinsichtlich der normativen Bewertung der Angebote der Sozialen Arbeit. Aus Forschungen zu den Nutzer*innen der Lebensmittelausgaben (Selke 2013; Schoneville 2013, 2017b, a) wissen wir, dass die Nutzung durch Widersprüche geprägt ist: Deutlich wird in den Berichten *erstens*, dass die Nutzer*innen auf die Hilfe angewiesen sind. Dies zeigt sich besonders in Berichten von Menschen, die die Angebote zunächst nicht wahrnehmen wollten, sich jedoch aufgrund der Verschlechterung ihrer materiellen Situation irgendwann gezwungen sahen, die Angebote zu nutzen. Die Nutzung erfolgt hier unter dem stummen Zwang der Armutsverhältnisse (Schoneville 2013). Unter diesen Bedingungen leisten die Lebensmittelausgaben, *zweitens*, durchaus eine bestimmte Form von Armutshilfe, welche von den Nutzer*innen als nützlich und hilfreich angesehen wird. Dass sich die Nützlichkeit auch hier vor dem Hintergrund der Armutssituation speist, wird etwa in Berichten deutlich, in denen die Nutzer*innen schildern, dass sie die Lebensmittelgabe wie ein Weihnachtsgeschenk erleben. Gleichzeitig zeigt sich, *drittens*, dass die Nutzer*innen Gefühle der Scham thematisieren. Die Schamgefühle beziehen sich dabei sowohl auf die konkrete Nutzung der Lebensmittelausgaben, die grundsätzliche Angewiesenheit auf eine solche Form der Hilfe als auch auf die spezifische gesellschaftliche Rolle, die damit eingenommen wird.

Eine zentrale Kritik an den „Tafeln“ aus einer armutstheoretischen Perspektive liegt darin, dass die Hilfsangebote die Menschen nicht dazu befähigen, sich aus der Armutssituation zu befreien. Sie leisten Hilfe nur insoweit, dass sie in einer Situation materiellen Mangels eine Form der Armutslinderung darstellen. Durch die Angebote der Lebensmittelausgabe werden die Nutzer*innen gesellschaftlich adressiert und damit auch in einer gewissen Weise integriert. Die Integration erfolgt jedoch nicht in Form einer Integration in den Konsummarkt für Lebensmittel oder eine Integration als Bürger*in durch staatsbürgerliche Rechte, sondern in Form einer mitleidsökonomischen Integration (Kessl et al. 2021), die als sekundäre Integration (Land und Willisch 2006; Kessl und Schoneville 2021) verstanden werden kann.

Vor dem Hintergrund der vorstehend vorgetragenen Analysen wurde in den vergangenen Jahren darauf hingewiesen (Kessl 2015; Groenemeyer und Kessl 2013; Kessl und Schoneville 2021), dass die Lebensmittelausgaben keineswegs singulär zu betrachten sind. Vielmehr hat sich ein System der Versorgung mit Elementargütern für von Armut betroffene Menschen herausgebildet, welches als „neue Mitleidsökonomie“ oder „Charity Economy“ bezeichnet werden kann. Die „neue Mitleidsökonomie“ steht dabei symptomatisch für die Abkehr von einer Politik der Armutsbekämpfung hin zu Hilfen, die auf Armutslinderung zielen.

### Kochkurse für Armutsbetroffene – Bildungsangebot oder Erziehung zur Armut?

Während die Hauptaktivität der Lebensmittelausgaben in der Verteilung von überschüssigen Lebensmitteln an von Armut betroffene Menschen liegt, legen einige „Tafeln“ Wert darauf, darüber hinaus auch andere Angebote bereitzustellen. Dazu gehören Treffpunkte, Cafés sowie auch Kochkurse. Aus sozialpädagogischer Perspektive erscheinen die Kochkurse als besonders interessant, verbinden sie doch die Hilfe in Bezug auf ein spezifisches soziales Problem mit einem Angebot, dass im Kontext von Erziehung und Bildung angesiedelt werden kann.

Auf der Webseite der Hamburger Tafel findet sich so zum Beispiel die nachfolgende Charakterisierung des eigenen Angebots^8^:

„Vor vielen Jahren hatte Annemarie Dose, die verstorbene Gründerin und Ehrenvorsitzende der Hamburger Tafel, die Idee, Kochkurse für jene Menschen anzubieten, die das Kochen noch nie gelernt oder schon wieder verlernt hatten. […]

Wir legen Wert darauf, dass die Kochkurse Menschen zusammenführen, die Interesse an gesunder, leckerer Ernährung haben. Außerdem bieten die Kochkurse den Menschen eine Plattform, auf der soziale Kontakte geknüpft werden können, wo man wegen der geleisteten Arbeit Respekt und Wertschätzung erfährt und die gemeinsame Mahlzeit am Ende des Kurses rundet diese Erfahrungen noch einmal positiv ab. […]

Obst und Gemüse, das die Teilnehmer*innen mit nach Hause nehmen können, sollen dafür sorgen, dass auch im Hause wieder selbst gekocht wird und die Tiefkühlpizza nur noch selten zum Einsatz kommt.“

In dem Statement werden die Teilnehmer*innen als eine Gruppe beschrieben, die entweder noch nie gekocht oder es verlernt hat. Ob dies tatsächlich so ist und in welchem Umfang dies zutrifft, lässt sich schwer einschätzen.^9^ Das Angebot insgesamt begründet sich in jedem Fall durch die Annahme, dass die fokussierte Gruppe in die Lage versetzt werden sollte, eigenständig zu kochen und der Kochkurs dazu beitragen könne. Bereits darin zeigt sich eine Spezifik des Angebots, schließlich waren Kochkurse in den vergangenen Jahren durchaus populär und wurden unter anderem von renommierten Restaurants und zu hohen Preisen angeboten. Diese gehen mitnichten davon aus, dass deren Kund*innen nicht kochen können, sondern sprechen Personen an, die den Kochkurs zum Beispiel als soziales Event nutzen oder aber auch hoch enthusiastische Hobbyköch*innen, die von Profis des Fachs lernen wollen.

Bemerkenswert erscheint, dass auf der oben zitierten Webseite keine Beschränkung des Teilnehmer*innenkreises auf armutsbetroffene Menschen vorgenommen wird. Bei der „Tafel“, wo entsprechende Bedürftigkeitsprüfungen durchgeführt werden, wurde dies immer wieder als stigmatisierend kritisiert. Man kann vermuten, dass die Anbieter*innen des oben genannten Angebots sich der Problematik einer solchen Einschränkung bewusst sind und durch inklusivere Formulierungen auch Stigmatisierungen entgegenwirken wollen. Zugleich wird jedoch mit dem Hinweis darauf, dass das Programm darauf ziele, dass die Menschen häufiger selber kochen und seltener „Tiefkühlpizza“ nutzen, auf ein Klischee zurückgegriffen, welches auch stigmatisierend sein kann.

Insgesamt zeigt sich hier die Ambivalenz dieser Form von Angeboten: Auf der einen Seite haben sie das Potenzial, als soziale und kulturelle Orte zu fungieren, die (auch) Menschen offenstehen, die sich teure Kochevents nicht leisten können. Sie können Menschen darin unterstützen, zentrale Kulturtechniken zu erlernen und dazu befähigen, ihre alltägliche Ernährung bewusster, abwechslungsreicher, gesünder und vielleicht auch kompetenter zu gestalten. Auf der anderen Seite können die Angebote aber gleichzeitig auch Stigmatisierungen aufrufen und verstärken. Sie unterliegen dabei der Gefahr, eine Problemverschiebung vorzunehmen und die Ursache für problematisch markierte Ernährung (ausschließlich) in fehlenden kulturellen Kompetenzen zu sehen und die eingeschränkten ökonomischen Möglichkeiten zu übersehen.

Lernen lässt sich hier von der bereits 15 Jahre zurückliegenden Debatte um die so genannte *neue Unterschicht*. Fabian Kessl et al. (2007) haben dabei davor gewarnt, dass Soziale Arbeit auch zu einer Form der „Erziehung zur Armut“ verkommen kann. In der Debatte wurde behauptet, dass das Problem der *neuen Unterschicht* eben nicht vornehmlich in ökonomisch-strukturellen Einschränkungen liege, sondern vor allem kultureller Natur sei. Es seien kulturelle Präferenzen der Menschen selbst, die zu Arbeitslosigkeit, Kriminalität, Teenagerschwangerschaften sowie falscher Ernährung führten und über Generationen weitergegeben werden. Zentraler Protagonist dieser Debatte war Charles Murray (1996), der Mitte der 1990er Jahre nach einem kurzen Aufenthalt in England einen Essay mit dem Titel „The Emerging British Underclass“ veröffentlichte. Für die Thesen ließen sich, jenseits der anekdotischen Erzählung, kaum Belege finden, und wissenschaftliche Untersuchungen (MacDonald und Marsh 2005) konnten die zentralen Aussagen widerlegen. Entsprechend wurde Murray dafür kritisiert, dass er eine Form von „blaming the victim“ (Walker 1996) betreibe. Dies verhinderte jedoch nicht, dass etwa zehn Jahre später ganz ähnliche Thesen von deutschen Journalist*innen hervorgebracht wurden. So hat Walter Wüllenweber im Stern zunächst unter dem Titel „Das wahre Elend“ (2004) und dann unter dem Titel „Voll Porno!“ (2007) Murrays Thesen öffentlichkeitswirksam neu aufbereitet. Auch hier sind es die kulturellen Präferenzen und Einstellungen, die als Grund für die als Elend und Verwahrlosung thematisierten Probleme ausgemacht werden. Schlechte Ernährung und mangelnde Hygiene spielen in dieser Erzählung eine große Rolle. Wirkmächtig wurde diese Debatte, die ohne empirische Evidenz geführt wurde, insbesondere auch, weil Kinder darin als zu schützende, der Kultur ihrer Eltern ausgelieferte Kinder thematisiert wurden. Während im wissenschaftlichen Diskurs deutliche Kritik formuliert wurde, gab es aus der Praxis der Sozialen Arbeit Zustimmung bis hin zu Beifall^10^. Dies mag zunächst verwundern, lässt sich aber unter anderem dadurch erklären, dass in der kulturalistischen Deutung von Armutsphänomenen Bildung und Erziehung eine zentrale Rolle eingeräumt wird.

In Bezug auf Kochkurse und ähnliche Angebote lässt sich aus dieser Debatte lernen. Sie stehen in der Gefahr, zum Teil eines solchen Diskurses zu werden, ihn zu verstärken und damit selbst Stigmatisierungen aufzurufen. Dies ist aber kein Automatismus. Die Kochkurse müssen nicht mit der Unterstellung eines kulturellen Defizits arbeiten und aus einer Position kultureller Überlegenheit angeboten werden, sondern können sich als Form allgemeiner kultureller Teilhabe verorten. Sie müssen sich nicht darauf konzentrieren, von Armut betroffenen Menschen die Fähigkeit zu vermitteln, mit den geringen Mitteln etwas besser auszukommen, sondern können zugleich auch Formen bereitstellen, in denen Interessen kollektiviert sowie artikuliert werden und so neue politische Teilhabeformen gefunden werden können.

## Armut als sozialpädagogisches Problem

Die Herausforderung, Essen und Armut aus einer sozialpädagogischen Perspektive zum Gegenstand zu machen, ist die gleiche Herausforderung, die in Bezug auf die Thematisierung von Armut durch die Sozialpädagogik insgesamt gilt: In welcher Weise können Phänomene der Armut sozialpädagogisch zum Gegenstand gemacht werden, ohne die Personen auf diesen Status festzuschreiben? Dies gilt hinsichtlich der Formen der Adressierung sowie vor allem in Bezug auf die zur Verfügung gestellten Formen der Hilfe und deren Ausmaß.

Angesichts der Veränderungen im Feld der Armutshilfen formulierten Fabian Kessl, Alexandra Klein und Sandra Landhäuser schon 2012, dass die Soziale Arbeit vor einer entscheidenden Wahl stünde: „Sie hat entweder die Möglichkeit, sich für die weitere Etablierung einer Mitleidsökonomie und vermehrter Disziplinierungsstrategien vereinnahmen zu lassen. Oder sie problematisiert im Sinne einer professionellen Reflexivität diese systematischen Verkürzungen und kämpft um alternative Perspektiven, deren Ziel eine Erweiterung oder zumindest Eröffnung von bisher nicht zugänglichen Handlungsmöglichkeiten der AdressatInnen sein sollte“ (Kessl et al. 2012, S. 546). Sozialpädagogische Hilfsangebote, sind aus dieser Perspektive solche, die den derzeitigen Status (als von Armut betroffene Menschen bzw. als Menschen, die Unterstützung benötigen) nicht festschreiben. Die Hilfe muss Menschen in die Lage versetzen, Bedingungen herzustellen, die die Möglichkeit enthalten, dass die jeweiligen Menschen ihre derzeitige Lebenssituation überwinden und in einer Art und Weise verändern können, die ihren eigenen Vorstellungen eines guten Lebens entsprechen.

Aus einer sozialpädagogischen Perspektive lassen sich so die Angebote gut begründet aus unterschiedlichen Richtungen befragen: Inwiefern können die Nutzer*innen die Angebote auch nicht nutzen? Welche Alternativen stehen zur Verfügung? Versetzen die Angebote die Menschen dazu in die Lage, ihre eigene Perspektive und Interessen zu artikulieren. Dies gilt sowohl innerhalb des institutionellen Settings der Angebote selbst wie auch für den öffentlichen Diskurs insgesamt. Schließlich stellt sich die Frage, ob die Angebote dazu in der Lage sind, die Menschen zu befähigen, sich aus der Armutslage zu befreien, um unabhängig von den Zwängen der Armutsbetroffenheit eigene Entscheidungen zu treffen. Normativ formuliert lassen sich damit Angebote kritisieren, die zum einen zwar notwendige Hilfen bereitstellen, zum anderen aber die Handlungsoptionen insgesamt nicht erweitern, sondern im Gegenteil neue Formen der Abhängigkeit schaffen und die Nutzer*innen auf ihren jeweiligen Status festschreiben.

Vor dem Hintergrund dieser normativen Verortung stellt sich im Rückblick auf die vier vorstehend diskutierten Angebotsformen, in denen die Soziale Arbeit auf Fragen von Armut und Essen reagiert, sowohl die Frage nach der systematischen Einordnung der Angebote, wie auch die Frage nach der professionspolitischen Positionierung zu ihnen.

Die integrierten regulären Essensangebote im pädagogischen Alltag wurden vorstehend bereits als unterschätzte Armutshilfen thematisiert. Sie sensibilisieren dafür, dass Angebote möglich sind, die nicht als Hilfen sichtbar werden. Sie können so gesellschaftliche Teilhabe für Kinder, Jugendliche und Erwachsene ermöglichen und gleichzeitig essensbezogene Angebote machen. Sie sind dabei nicht auf armutsbetroffene Jugendliche fokussiert. Dies kann zum einen als Stärke bewertet werden, weil Menschen damit gar nicht als Armutsbetroffene adressiert und sichtbar werden. Zum anderen kann kritisiert werden, dass die Angebote wenig zielgenau sind. Das Potenzial dieser Form von Essensangebot wird jedoch unterlaufen, wenn die Angebote kostenpflichtig sind und von Armut betroffene Menschen sich Bedürftigkeitsprüfungen unterziehen müssen, um daran kostenlos oder vergünstigt teilzunehmen. In ähnlicher Weise gilt dies, wenn die (schlechte) Qualität dieser Angebote dazu führt, dass nur Menschen dieses nutzen, die dazu aufgrund ihrer materiellen Situation gezwungen sind.

Angebote der Nothilfe, wie sie anhand der Suppenküchen diskutiert wurden, stehen im Vergleich zu den integrierten, regulären Essensangeboten am anderen Ende des Spektrums. Sie zielen explizit darauf, Menschen zu unterstützen, deren Existenz in ihrem Alltag fundamental gefährdet ist – wie dies bei Obdach- oder Wohnungslosigkeit der Fall ist. Hier steht die existenzielle Sicherung im Vordergrund. Nothilfe alleine ist jedoch keine ausreichende Unterstützung, um Menschen in die Lage zu versetzen, die Armutssituation zu überwinden. Sie kann bestimmte existenzielle Bedrohungen abmildern und damit die Basis für andere Handlungsmöglichkeiten bieten. Aus diesem Grund erscheint die Forderung begründet, dass die Nothilfe nicht bei der Nothilfe stehen bleiben kann, sondern weiterführende Beratungsangebote sowie Formen der politischen Einmischung beinhalten muss.

Die „Tafeln“ und ähnliche Angebote wurden ebenfalls, vor allem zu Beginn, als Nothilfe beschrieben. Die Struktur ihrer Angebote zeigen jedoch, dass sie vielmehr eine Form der mitleidsökonomischen Armutslinderung darstellen. Die Angebote der „Tafeln“ werden unter den bestehenden Bedingungen von den Nutzer*innen benötigt. Darauf verweisen nicht nur die Nutzungszahlen, sondern auch die empirischen Erkenntnisse, die zu den Nutzer*innen vorliegen. Sie leisten in diesem Sinne eine Form der Unterstützung, gleichzeitig sind sie nicht in der Lage, Menschen dazu zu befähigen, die Armutssituation zu überwinden. Vielmehr lassen sich die Angebote aus einer ausgrenzungstheoretischen Perspektive selbst als Form der sekundären Integration oder als Integration unter den Bedingungen von Ausgrenzung beschreiben (Kessl und Schoneville 2021). In den damit aufgerufenen Konzepten werden zum einen die Integrationsleistungen der Hilfen Rechnung getragen, zum anderen jedoch wird darauf verwiesen, dass die Versorgung durch eine „Tafel“ weiterhin unter den Bedingungen von Ausgrenzung stattfindet. Die wohlfahrtsstaatstheoretische Einordnung verdeutlicht, dass die „Tafeln“ und ähnliche Angebote zu einem Teil des wohlfahrtsstaatlichen Arrangements geworden sind. Die Gestalt des wohlfahrtsstaatlichen Arrangements ist immer auch als das Resultat von politischen Entscheidungen zu verstehen. Insofern lassen sich die mitleidsökonomischen Angebote der „Tafel“ im Kontext einer sozialpolitischen Instrumentalisierung (bzw. Indienstnahme siehe Boemke et al. [2021]) beschreiben und kritisieren, mit der Formen der Armutslinderung – und nicht der Armutsbekämpfung – etabliert wurden.

Die bildungsbezogenen Essensangebote, wie sie anhand der Kochkurse diskutiert wurden, können neue Handlungsoptionen öffnen, insofern sie Orte der sozialen und kulturellen Teilhabe sind, schließlich ist Essen und Kochen immer auch mehr als nur physische Versorgung. Sie können zudem Handlungsoptionen eröffnen, indem sie Wissen und Kulturtechniken rund ums Kochen vermitteln. Diese bildungsbezogenen Angebote sind jedoch nicht in der Lage dazu, die materielle Situation grundlegend zu verändern und stehen zugleich in der Gefahr, das Problem der Armut zu individualisieren und zu kulturalisieren. Dies wäre der Fall, wenn ernährungsbezogene Armutsprobleme mit Verweis auf mangelndes Wissen und zu geringe Erfahrungen im Kochen erklärt werden und die Frage der materiellen Möglichkeiten ignoriert wird.

Politische Positionierungen der Sozialen Arbeit sind vor dem Hintergrund der vorstehend genannten Diskussion nicht nur notwendig, weil sich die Armutsbetroffenheit ausweitet und die Lebensrealitäten von armutsbetroffenen Menschen sich weiter zuspitzen. Sie sind auch in Bezug auf die Hilfen im Feld der Sozialen Arbeit notwendig. Dies gilt sowohl nach außen – zum Beispiel hinsichtlich der Instrumentalisierung von freiwilligen Hilfen durch staatliche Sozialpolitik – als auch nach innen. Schließlich sind es Akteure der Sozialen Arbeit, die Hilfen anbieten, die aus sozialpädagogischer Perspektive nicht nur nicht begründbar sind, sondern kritisiert werden müssen. Dies gilt zumindest dann, wenn der Anspruch der Sozialen Arbeit lautet, die Handlungsoptionen ihrer Adressat*innen erweitern zu wollen und einen Beitrag zur Bekämpfung von Armut zu leisten.

## Footnotes

1 https://www.tagesspiegel.de/politik/die-armsten-sollten-sich-nicht-entscheiden-mussen-4348561.html (Abruf: 30.10.2022) https://www.ndr.de/fernsehen/sendungen/panorama3/Arm-durch-Inflation-Wie-Preissteigerungen-zum-Risiko-werden,inflation154.html (Abruf: 30.10.2022) https://www.merkur.de/wirtschaft/rente-rentner-inflation-alter-armut-energiekrise-preise-heizkosten-grundsicherung-muenchen-aktuell-91792510.html (Abruf: 30.10.2022)

2 Diese Daten beruhen auf dem sozioökonomischen Panel. Die Daten des Mikrozensus sowie des EU-SILC unterscheiden sich etwas, weisen im Wesentlichen aber ähnliche Werte auf.

3 Sozialgesetzbuch, Zweites Buch, § 20 Regelbedarf zur Sicherung des Lebensunterhalts.

4 Mit der nunmehr beschlossenen Einführung des Bürgergelds zum 01.01.2023 ist eine Erhöhung der Regelsätze auf insgesamt 502 €/Monat für eine alleinstehende Person vorgesehen (Bundesministerium für Arbeit und Soziales 2022). Vom Paritätischen Wohlfahrtsverbands (2022) wird diese jedoch kritisiert, da die Erhöhung unterhalb der Inflationsrate bleiben wird.

5 Stephan Lorenz (2012) hat dabei eindrücklich gezeigt, dass die Lebensmittelausgaben – mindestens rhetorisch – mit dem Passungsverhältnis zwischen dem Überfluss an Lebensmittel auf der einen Seite und dem Mangel an Lebensmitteln auf der anderen Seite operieren.

6 Die Diskrepanz ergibt sich aus unterschiedlichen Berichten durch den Bundesverband. Dieser hatte im Sommer 2022 in einem Update berichtet, dass es über 2 Mio. Nutzer*innen seien. Im August wiesen die regelmäßig aktualisierten Hintergrundinformationen 1,65 Mio. Nutzer*innen aus. Die Diskrepanz kann daraus resultieren, dass die Zahlen auf Selbstauskünften der lokalen „Tafeln“ beruhen und dann hochgerechnet werden. Eine andere Erklärung wäre auch, dass die „Tafeln“ für Geflüchtete aus der Ukraine, eine zentrale erste Hilfestelle waren. Diese Lesart legt das Update zur Situation aus dem Sommer 2022 nahe, da dort erhebliche Steigerungen der Nutzungszahlen durch geflüchtete Menschen aus der Ukraine ausgewiesen werden.

7 Die von Markus Grabka und Jürgen Schrupp präsentierte DIW-Studie ist bemerkenswert: Bislang basierten Zahlen zu den Lebensmittelausgaben fast ausschließlich auf Selbstauskünften von Tafel Deutschland. Die DIW-Studie basiert nun auf Zahlen des sozioökonomischen Panels, in dem einige Fragen zur Nutzung der „Tafeln“ aufgenommen wurden. Da das SOEP eine repräsentative Längsschnittstudie darstellt, ergeben sich ganz neue Möglichkeiten für die Armutsforschung in Bezug auf Fragen der Nutzung von Hilfsangeboten sowie der Bewältigung von Armut. [Kudos, DIW!]

8 https://hamburger-tafel.de/was-wir-tun/kochkurse-kochbuch/ (Abruf: 30.10.2022)

9 Eine wissenschaftliche Auseinandersetzung mit diesen Angeboten steht noch weitgehend aus. Die bislang vorliegenden empirischen Beobachtungen scheinen sich auf die Selbstevaluation einiger Programme zu beschränken (Schnellbächer et al. 2017).

10 So hat Walter Wüllenweber im Jahr 2005 für seinen im Dezember 2004 im Stern veröffentlichten Text „Das wahre Elend“ den Deutschen Sozialpreis erhalten. Dieser wird immerhin von der Bundesarbeitsgemeinschaft der Freien Wohlfahrtspflege vergeben. https://www.bagfw.de/ueber-uns/deutscher-sozialpreis/archiv/preistraeger-seit-1971 (Abruf: 30.10.2022)
